# Pattern of fin rays along the antero-posterior axis based on their connection to distal radials

**DOI:** 10.1186/s40851-019-0145-z

**Published:** 2019-09-18

**Authors:** Hiroki Hamada, Toshiaki Uemoto, Yoshitaka Tanaka, Yuki Honda, Keiichi Kitajima, Tetsuya Umeda, Atsushi Kawakami, Minori Shinya, Koichi Kawakami, Koji Tamura, Gembu Abe

**Affiliations:** 1 Laboratory of Organ Morphogenesis, Department of Ecological Developmental Adaptability Life Sciences, Graduate School of Life Sciences, Tohoku University, Sendai, 980-8578 Japan; 2 School of Life Science and Technology, Tokyo Institute of Technology, Yokohama, 226-8501 Japan; 3 Department of Biology, Keio University, Yokohama, 223-8521 Japan; 4 Laboratory of Molecular and Developmental Biology, National Institute of Genetics, Mishima, Shizuoka, 411-8540 Japan; 5 Department of Genetics, SOKENDAI (The Graduate University for Advanced Studies), Mishima, Shizuoka, 411-8540 Japan

**Keywords:** Fin ray, Phenotypic variation, Zebrafish strains, Antero-posterior patterning, IM-II

## Abstract

**Background:**

Teleost paired fins are composed of two endoskeletal domains, proximal and distal radials, and an exoskeletal domain, the fin ray. The zebrafish pectoral fin displays elaborately patterned radials along the anteroposterior (AP) axis. Radials are considered homologous to tetrapod limb skeletons, and their patterning mechanisms in embryonic development are similar to those of limb development. Nevertheless, the pattern along the AP axis in fin rays has not been well described in the zebrafish pectoral fin, although several recent reports have revealed that fin ray development shares some cellular and genetic properties with fin/limb endoskeleton development. Thus, fin ray morphogenesis may involve developmental mechanisms for AP patterning in the fin/limb endoskeleton, and may have a specific pattern along the AP axis.

**Results:**

We conducted detailed morphological observations on fin rays and their connection to distal radials by comparing intra- and inter-strain zebrafish specimens. Although the number of fin rays varied, pectoral fin rays could be categorized into three domains along the AP axis, according to the connection between the fin rays and distal radials; additionally, the number of fin rays varied in the posterior part of the three domains. This result was confirmed by observation of the morphogenesis process of fin rays and distal radials, which showed altered localization of distal radials in the middle domain. We also evaluated the expression pattern of *lhx* genes, which have AP patterning activity in limb development, in fin rays and during distal radial development and found these genes to be expressed during morphogenesis in both fin rays and distal radials.

**Conclusion:**

The fin ray and its connection to the endoskeleton are patterned along the AP axis, and the pattern along the AP axis in the fin ray and the radial connection is constructed by the developmental mechanism related to AP patterning in the limb/fin bud. Our results indicate the possibility that the developmental mechanisms of fin rays and their connection are comparable to those of the distal element of the limb skeleton.

**Electronic supplementary material:**

The online version of this article (10.1186/s40851-019-0145-z) contains supplementary material, which is available to authorized users.

## Introduction

Vertebrates have highly elaborated locomotor appendages, namely, fins and limbs, which are adapted to their habitats. The skeletal patterns of locomotor appendages are highly diverse, providing ample opportunities to study the evolutionary mechanisms underlying morphological diversity [1, 2].

**Fig. 1:**
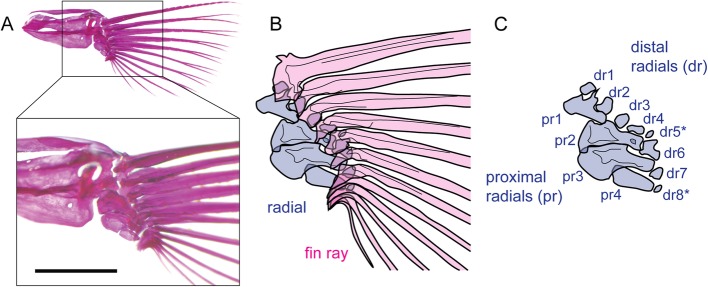
Skeletal anatomy of the zebrafish pectoral fin. **a** Skeletal anatomy of the zebrafish pectoral fin with Alizarin Red staining. The bottom box shows a magnified view of the upper whole pectoral fin skeleton in the inset. The scale bar indicates 1 mm. **b**, **c** Schematics of the fin rays and radials. Drawings in dark blue and pink indicate radials and fin rays, respectively. Each of the proximal radials (pr) and distal radials (dr) are indicated in **c**. dr5, and dr8 occasionally did not appear (asterisk)

Teleost paired fins are composed of two endoskeletal domains, the proximal and distal radials, and an exoskeletal domain, the fin ray, and the zebrafish pectoral fin is a representative appendage among teleost species, from both a morphological and developmental biological perspective. The zebrafish pectoral fin has four proximal radials aligned along the anteroposterior (AP) axis that are articulated distally to 6–8 radials (Fig. 1a-c) [3]. In these radials, the proximal radial skeletons develop from a primordial endochondral disc, which appears in the fin bud at 2 dpf and later subdivides into each proximal radial at approximately three weeks post-fertilization (3 wpf) [3–5]. The distal radials appear distal to the proximal radials after subdivision of the endochondral disc [3]. At the same time, mesenchymal cells migrating into the apical fold (AF) form the fin rays. These fin rays are adjacent to the distal radials at their proximal ends, but the topological relationship between fin rays and distal radials remains obscure [3, 4]. After several anterior fin rays have formed, the first to fourth distal radials appear at the base of the fin rays [3], but the topological relationship among the posterior fin rays and distal radials and their developmental process remains unclear [3]. Moreover, the number of fin rays in zebrafish ranges from 10 to 14 [3]. Thus, it is possible that the fin ray developmental process and topological relationship to distal radials vary highly. As discussed below, elaborately patterned endoskeletons are formed by the mechanism of pattern formation shared with that of limb endoskeletons, but exoskeletons in the fin may be regulated in part by stochastic self-organization mechanisms.

Proximal and distal radials, i.e., the fin endoskeleton, are considered homologous to the tetrapod limb endoskeleton [6, 7], and the process and mechanism of their patterning in embryonic development are similar to those of tetrapod limb development. During development, both paired fin radials and the limb endoskeleton are structured from the fin/limb bud containing two signaling centers, the apical ectodermal ridge (AER) and the zone of polarizing activity (ZPA), which are located at the dorsoventral border of the ectoderm and in the posterior mesenchyme, respectively [7–10]. The AER and ZPA secrete signaling molecules, and direct expression of patterning genes and establish morphology along the PD and AP axes in the fin/limb bud. Although paired fin radials lack some structures corresponding to the autopod domain in the limb [6, 7], they share key molecules and basic mechanisms for patterning.

Fin rays develop in the AF, into which the AER transitions at a late embryonic stage [3, 11, 12]. In addition, this AER-to-AF transition, which may cause depletion of AER function in fin bud development, is considered a key feature of fin-to-limb evolution [7, 12, 13]. Several recent reports have suggested pattern formation mechanisms of mesenchymal cells in the fin fold at the early larval stage and that these mechanisms are evolutionarily and developmentally related to the above-described mechanisms for formation of the endoskeletal parts of fins and limbs. The lateral plate mesoderm is the developmental origin of the fin ray skeletons in the pectoral fin and is also the origin of the endoskeletal domain in pectoral fins and limbs [14]. Furthermore, some transcription factor genes for endoskeletal pattern formation, such as *hox* and *lhx* genes, are expressed in precursor cells of the pectoral fin ray region at an early larval stage [15–19]. Therefore, fin ray morphogenesis at a later larval stage may involve similar developmental mechanisms for AP patterning in the fin/limb bud. If these mechanisms apply, do fin rays have a certain pattern along the AP axis?

Here, we show that pectoral fin ray skeletons can be categorized into three domains along the AP axis according to the mode of connection between fin rays and distal radials and that the number of fin rays varies in the posterior part of the three domains. We also examined the process of morphogenesis of fin rays and distal radials, which were visualized in live fish using chondrogenesis reporter fish and skeletal staining. Furthermore, we assessed specific reporter activity of *lhx* genes in developing fin rays and distal radials. Based on our results, fin rays and their connection to the endoskeleton are patterned, and the pattern is constructed by a developmental mechanism related to fin/limb bud patterning along the AP axis.

## Materials and methods

### Fish strains

Adult zebrafish were collected from among RIKEN wildtype (RW) (from RIKEN BioResource Center for zebrafish), Tubingen (from Tohoku University, the Ogura Laboratory), TAB and AK (from National Institute of Genetics, the Kawakami Laboratory), and IM-II (from Keio University, the Shinya Laboratory) zebrafish. TAB is an AB-background fish crossed with TL and maintained over successive generations. AK is a progeny from a wild population collected in North India [20] and maintained over ten generations in the Kawakami Laboratory fish facility. IM-II was inbred over 20 generations in the National Institute of Genetics and then branched at generation 29 for inbreeding in the Shinya Laboratory [21]. A sample commercially purchased from a local aquarium store (WP) was also used. The following transgenic lines were used in this study: *sp7:mcherry* [22], *gt1641A* (*gSAIzGFFD1641A:gal4*), *gt223A* (*SAGFF(LF)223A:gal4*), *UAS:GFP* [23], and *col2a1a:EGFP*. To identify the integration site for *gal4ff* in the *gt1641A* and *gt223A* lines, Southern blotting and inverse polymerase chain reaction (PCR) were performed as previously described [24]. To generate *col2a1a:EGFP*, we used a genomic fragment of the *collagen, type II, alpha 1a* (*col2a1a*) gene [25]. The genetic background of transgenic fish is a hybrid of AB and TL. The standard length (SL) of each fish was measured, and the zebrafish were maintained according to standard protocols [26]. All experimental animal care was in accordance with institutional and national guidelines and regulations and was approved by the Tohoku University Animal Research Committee (Permit Number: 2018LsA-015).

### Skeletal staining of the adult pectoral fin

Fish were euthanized with 0.1% MS-222 (Tokyo Chemical Industry), and the pair of pectoral fins was dissected. The fins were fixed with 10% formalin in distilled water (DW) overnight and dehydrated in an ethanol/DW series (50% ethanol/DW, 70% ethanol/DW, 80% ethanol/DW, 90% ethanol/DW, 95% ethanol/DW) for 20 min in each solution and then placed in 100% ethanol overnight. After dehydration, the fins were stained overnight (modified from Walker and Kimmel, 2007; Part A solution (10 mM MgCl_2_ and 0.2% alcian blue in 70% ethanol/DW) and Part B solution (0.5% Alizarin Red in DW) mixed at a ratio of 12.5:1). The tissues were bleached and cleaned overnight in 0.5% KOH and 50% glycerol, 0.25% KOH and 50% glycerol, and 50% glycerol. The skeletal morphology of the pectoral fins was analyzed in a 50% glycerol solution under a stereoscopic microscope (Olympus SZX16).

### Alizarin red staining of the larval pectoral fin ray

Vital staining with Alizarin Red solution was performed as previously described [27], with minor modifications. In brief, 0.005% Alizarin Red in system water was prepared. Zebrafish larvae were transferred to the Alizarin Red solution and kept in the solution for 30–60 min. After staining, the larvae were rinsed in system water several times.

### Microscopic analyses of the larval pectoral fin

Zebrafish larvae were anesthetized with MS222 and then embedded in 2% methyl cellulose/E3 on a slide glass and analyzed using a Leica M205 FA microscopic system with a Leica DFC 369 FX camera. Images were obtained and analyzed with Leica LAS-AF and Adobe Photoshop CS4. After observation, the larvae were immediately transferred to a small case filled with system water [26] and awakened by sprayed water.

## Results

### Skeletal anatomy of pectoral fin rays in laboratory strains and noninbred wildtype zebrafish

**Fig. 2:**
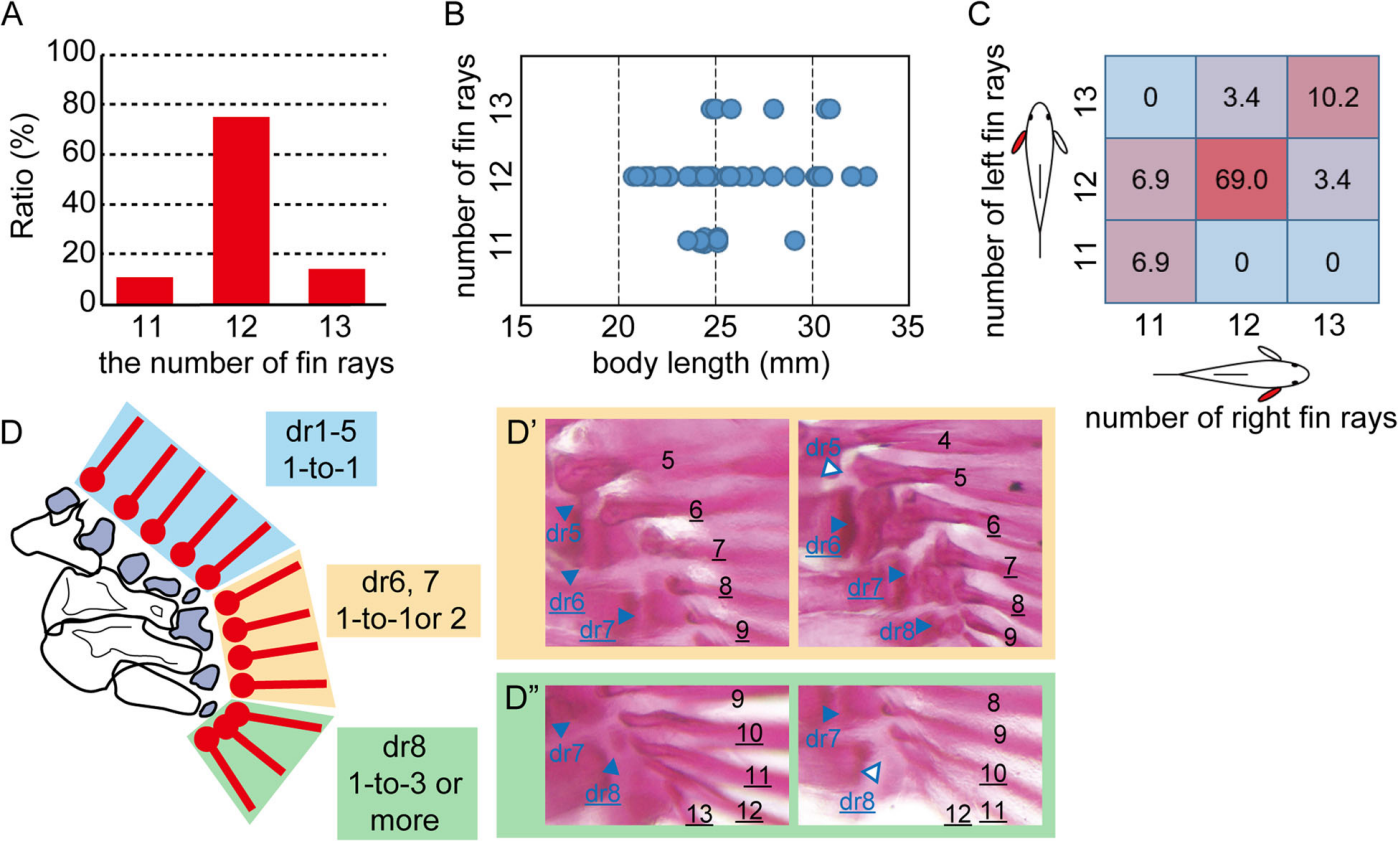
Observation of skeletal anatomy of the pectoral fin in the RW strain**. a** Distribution of the variation in fin ray number. **b** Relationship between the number of rays and body length. Blue circles indicate individuals. **c** Ratio of the combination of the numbers of fin rays in the left and right pectoral fins. **d**. Schematic drawing of the connection between the fin rays (red bar with circle) and distal radials (dark blue). Light blue, orange, and green boxes indicate the relationship in the one-to-one, one-to-one or two, and one-to-three or more modes, respectively. **d’**. Connection between fin rays and the sixth and seventh radials. The seventh radial showed connection in a one-to-two mode; the sixth radial showed the connection in a one-to-two (left panel) or a one-to-one (right panel) mode. **d”**. Connection between the fin rays and the eighth radial. The number of fin rays connected to the eighth radial varied (four rays in the left panel and three rays in the right panel). Blue-colored arrowheads with dr5–8 indicate the 5th to 8th distal radials, respectively. Blue-outlined arrowheads with dr5 in the right panel of **d’** and dr8 in the right panel of **d”** indicate the absence of the 5th and 8th distal radials, respectively. Black-colored numbers indicate fin ray numbers. Underlined characters in **d’** indicate the 6th and 7th distal radials and the fin rays connected to them. Underlined characters in **d”** indicate the 8th distal radial and the fin rays connected to it

In the zebrafish pectoral fin, the number of fin rays varies among individuals [3]. Thus, we first examined skeletal morphology in the zebrafish pectoral fin with regard to how the variation appears in the fin ray and its topological relationship to distal radials. We examined the fin ray in the RW strain, observing 56 pectoral fins from 29 RW fish with Alizarin Red skeletal staining. The total number of fin rays of each pectoral fin varied from 11 to 13 (11 rays: *n* = 6, 12 rays: *n* = 43, 13 rays: *n* = 9) (Fig. 2a). Furthermore, the variation in fin ray number did not correlate with the specimen’s body size (Fig. 2b). In addition, the numbers of rays occasionally differed on the left and right sides of the same individual (13.7%), though the majority of specimens had the same number of rays on both sides (Fig. 2c).

**Fig. 3:**
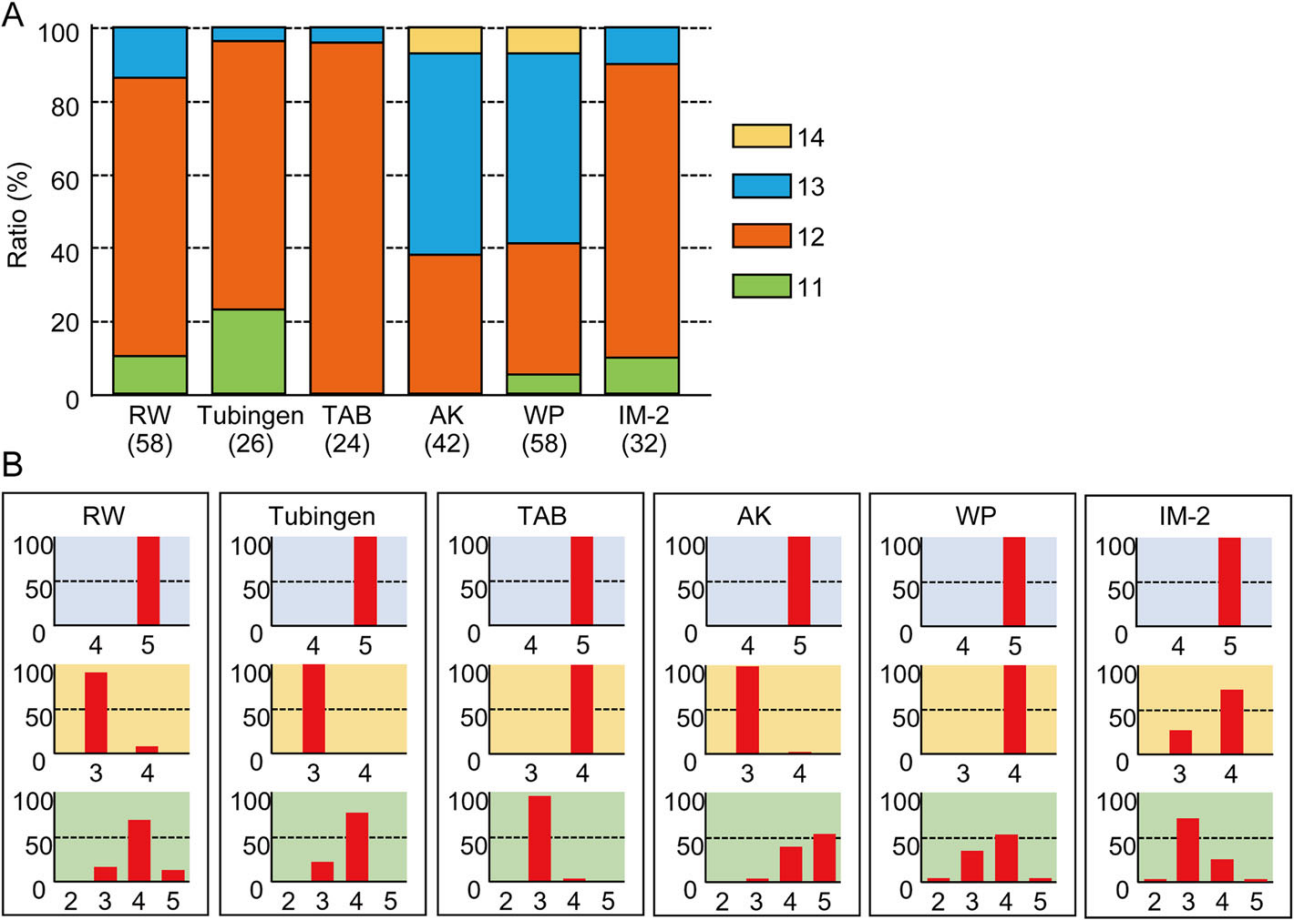
Variation in the number of fin rays within the three domains of the pectoral fin of zebrafish strains. **a** Proportion of specimens with different numbers of fin rays among the zebrafish strains. The number of specimens analyzed is indicated under the strain’s name. **b** Relationships between the number of fin rays and fin domains. Graphs colored light blue, orange, and green are in the anterior (dr1–5), middle (dr6, 7), and posterior (dr8) domains. Numbers on the vertical axis of graphs indicate the ratio (%) of the distribution of specimens. Numbers on the lateral axis indicate the number of fin rays in the domains. Red bars indicate the ratio of specimens to the number of fin rays

Regarding variation in the number of pectoral fin rays, we examined whether the fin rays are placed randomly or in an orderly manner in the pectoral fin. A fin ray is adjacent to a distal radial(s) at its proximal base (Fig. 1). Based on this topological relationship, we estimated which fin ray is connected to which distal radial in the RW fish pectoral fins. As previously described [3], the most anterior four fin rays were connected to the four anterior distal radials in one-to-one correspondence (Fig. 2d). The anterior fifth fin ray also exhibited one-to-one connection with the fifth distal radial (Fig. 2d and d’’), though the fifth radial was occasionally absent; in such a case, the base of the fifth fin ray was located where the fifth radial would have been (Fig. 2d’, right panel). One or two fin rays were connected to the sixth radial (Fig. 2d and d’). Two fin rays were always connected to the seventh radial (Fig. 2d and d’). Alternatively, three to five fin rays were connected to the eighth radial (Fig. 2d, d”). The eighth distal radial was occasionally invisible, as was the fifth radial (Fig. 2d”, right panel). Based on these results, we categorized the modes of connection between the fin rays and distal radials into three domains: the anterior domain for the five anterior distal radials with one-to-one connection, the middle domain for the sixth and seventh distal radials with one-to-one or one-to-two connection, and the posterior domain for the eighth radial with one-to-more than three connection (Fig. 2d). Regarding the distribution of variation, there was no variation in fin ray numbers (always five fin rays) in the anterior domain (Fig. 3b, top blue graph of the RW column), and over 90% of specimens had a fin ray at the sixth radial and a total of three fin rays at the middle domain (Fig. 3b, middle orange graph of the RW column). In the posterior domain, the number of fin rays varied from three to five, and 71.4% of the specimens had four fin rays (Fig. 3B, bottom green graph of the RW column).

Next, to determine whether the categorization of connections is a common feature among zebrafish strains, we evaluated the fin ray and distal radial connection in other laboratory strains, i.e., Tubingen, TAB, and AK, and in a commercially purchased pet-shop zebrafish (WP) (Fig. 3). Tubingen is a well-known zebrafish wildtype strain used in mutagenesis studies [28, 29]. TAB is a progeny of two notable wildtype strains, AB and TL, purified with a stripe pigment pattern and without a long-fin phenotype [30]. AK is a progeny from a wild population collected in North India [20] that had been maintained over ten generations under laboratory conditions. Similar to the RW strain, the Tubingen, TAB, AK and WP strains display variation in the number of pectoral fin rays (Fig. 3a). Tubingen and TAB mainly had 12 fin rays, and approximately 55% of AK and WP had 13 fin rays; most of others had 12 fin rays. We also examined the distribution of fin rays in the three domains and observed no variation in the five anterior fin radials, and all of the specimens had 5 fin rays in one-to-one correspondence (Fig. 3b, upper blue graphs). In the middle domain with the sixth and seventh distal radials, the distribution of fin rays varied among the strains within the range of one or two (Fig. 3b, middle orange graphs). At the posterior eighth radial, the number of fin rays varied among the strains (Fig. 3b, bottom green graphs). The number of fin rays at the posterior region in Tubingen and TAB was three or four. In AK and WP, the number of fin rays sometimes reached five. WP showed maximum variation with two to five rays. Although the distributions of the number of fin rays in the middle and posterior regions differed among the strains (Fig. 3b), the results suggest that the categorization of connections is common among these zebrafish strains.

### Environmental effects on variation in the number of fin rays

Because zebrafish strains have strain-dependent genetic diversity and this diversity is related to differences in biological responses among strains [31, 32], the difference in the numbers of fin rays among the strains assessed might be explained to some extent by genetic diversity. However, some individuals had different numbers of fin rays in the pectoral fins on the left and right sides (Fig. 2b), suggesting that some nongenetic factors, including developmentally stochastic events and environmental stimuli, also affect variation in fin ray anatomy. To determine the degree to which non-genetic factors contribute to this variation, we examined the skeletal anatomy of the pectoral fin in individuals sharing, as much as possible, the same genetic background. For this purpose, we used the IM-II strain, which was inbred for 34 generations [21]; it is expected to have a lower amount of genetic polymorphism than other laboratory strains. Based on morphological observations, the IM-II strain mainly exhibited 12 pectoral fin rays but also showed variation in the number of fin rays (Fig. 3a, IM-II column). Moreover, we observed variations in the number of fin rays in the middle and posterior domains in the same manner as in other strains (Fig. 3b, IM-II column). These results suggest that intra-strain variation, such as variation in the number of fin rays within the posterior part, may be greatly affected by non-genetic factors.

### Process of morphogenesis of the pectoral fin rays and distal radials

Because the variation in the number of fin rays did not correlate with the difference in body size of adult fish (Fig. 2b), the number of fin rays and their distal radial connection might be determined at a certain stage of pectoral fin development. Accordingly, we examined how fin rays and distal radials appear during pectoral fin morphogenesis. To observe this process, we used individual tracing with transgenic and fluorostained live fish using *col2a1a:EGFP* to visualize the cartilage of radials [25] and Alizarin Red staining for calcified fin ray bone in live fish [27] or *sp7:mcherry* transgenic fish for fin ray osteoblast cells [22].

**Fig. 4:**
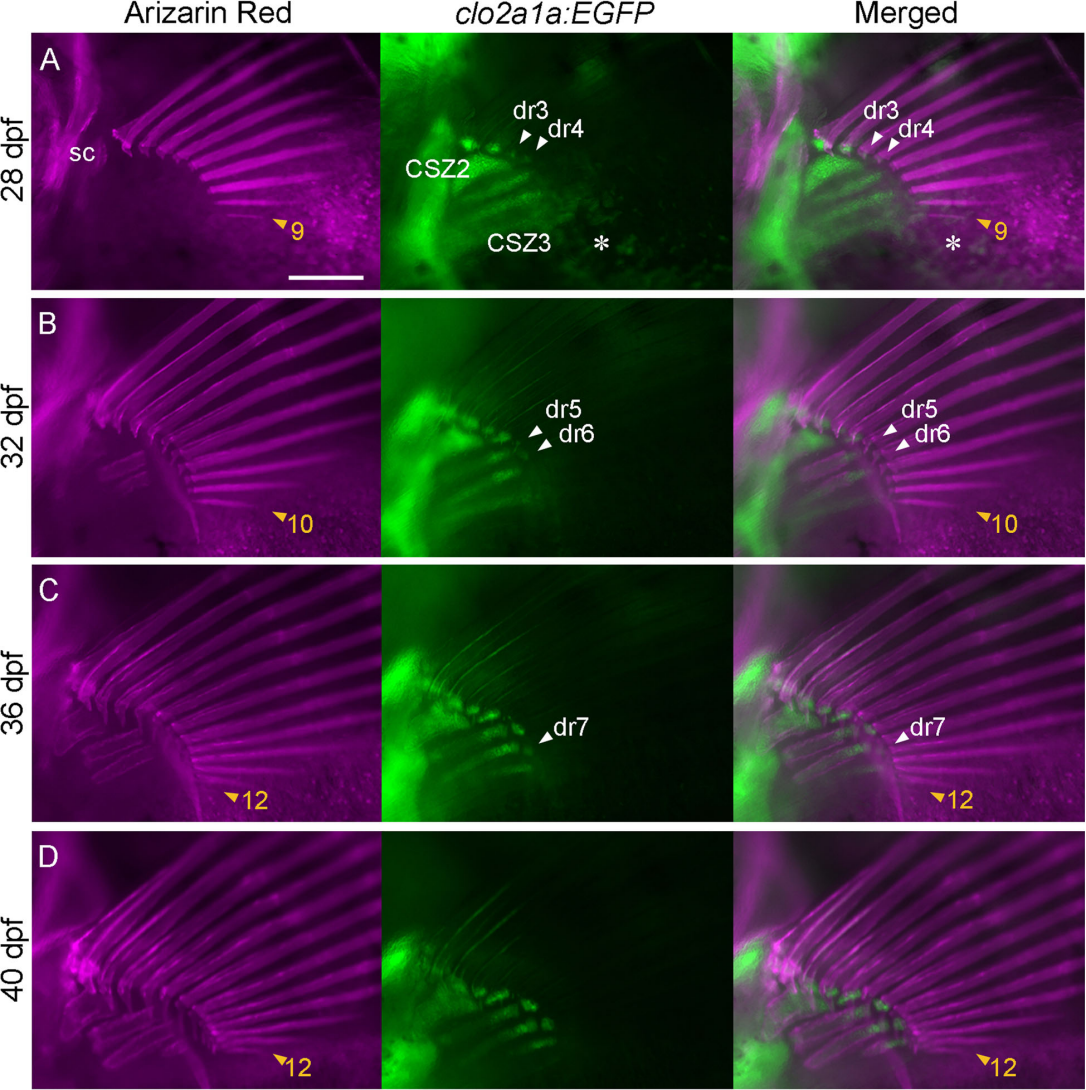
Process of morphogenesis of the fin rays and radials. Calcified bones (Alizarin Red) and chondrogenic cells (*col2a:EGFP*) of the left pectoral fin in individual tracing specimens were observed at 28 dpf (**a**), 32 dpf (**b**), 36 dpf (**c**), and 40 dpf (**d**). The observed fish were crossbred. Orange arrowheads with numbers indicate the most posterior fin ray. White arrowheads with dr3–7 indicate newly appearing distal radials. The scale bar in **a** indicates 200 μm. CSZ, cartilage subdivision zone. sc, anlagen of the scapula. The asterisk in **a** indicates the reflected signal of the iridophore on the swim bladder

As previously reported, several anterior fin rays develop before the appearance of distal radials at 21 dpf; at 24 dpf, two or three distal radials appear, and three or four additional fin rays develop (Additional file 1) [3]. Hence, we started individual tracing at 24 dpf (*n* = 3; SL = 7.6–7.8 mm) and observed specimens every 4 days until 44 dpf and at several time points after 44 dpf (Fig. 4; Additional file 2 and data not shown). A specimen that finally had 13 fin rays is shown in Fig. 4. During the observation period, the number of fin rays increased from 6 to 13, in order from anterior to posterior (Additional file 2A–F). At 36 dpf, the 12th fin ray could be observed (SL = 9.5 mm) (Fig. 4d), and the number of fin rays remained unchanged at 12 to 44 dpf (SL = 10.7 mm) (Additional file 2F). Nevertheless, at 54 dpf, we found a 13th fin ray in one specimen (SL = 12.0–12.8 mm). However, the number did not change at later stages, including in other specimens (Additional file 2G and data not shown), suggesting that the number of fin rays was determined at a time between 44 and 54 dpf.

In the formation of fin rays and their connection to distal radials, the first to fourth distal radials appeared at the base of the fin rays in a one-to-one manner until 28 dpf, as previously reported (Fig. 4a; Additional file 2A, B) [3, 4]. The fifth and sixth distal radials also appeared at the base of the fifth and sixth fin rays at 32 dpf (*n* = 3) (Fig. 4b). The size of the sixth distal radial increased as the size of the anterior distal radials increased at 36 dpf, whereas the size of the fifth distal radial did not increase (Fig. 4b, c). Alternatively, the seventh distal radial appeared at the base of the seventh and eighth fin rays at 36 dpf (Fig. 4c). The eighth radial also appeared at the base of the ninth to thirteenth fin rays at 54 dpf (Additional file 2G). These results suggest that the mode of connection was altered from one-to-one to one-to-two or more at the time of the appearance of the seventh distal radial, corresponding to morphological observation of the adult pectoral fin skeletal pattern (Fig. 2). Notably, the 7th and 8th distal radials were adjacent to the distal tip of the 3rd and 4th proximal radials at the beginning of their appearance, respectively (Fig. 4c; Additional file 2G). However, the second to fourth distal radials and the sixth distal radial appeared to connect the first and second proximal radials appearing at 32 dpf and 36 dpf, respectively (Fig. 4b, c; Additional file 2A–D); in contrast, the fifth distal radial did not appear to connect the proximal radials. These results indicate that because the distal radials became connected to the proximal radials after approximately 32 to 36 dpf, the anterior distal radials began to form at the base of the fin rays without association with the proximal radials but that the posterior distal radials connected to the proximal radials from the beginning. These findings suggest that alterations in this mode of localization resulted in the transition of the connection mode of the fin ray and distal radial relationship from one-to-one to one-to-many.

### Expression of limb developmental genes during the formation of fin rays and distal radials

Because the fin rays and their distal radial connection showed a well-ordered morphology along the AP axis, a discrete developmental mechanism may play a role in the morphogenetic process. In addition, based on previous research, precursor cells of the distal radials and fin rays at an early larval stage share some molecular properties with the developing limb bud [15–19]. We hypothesized that the formation of fin rays and distal radials at a later larval stage may be driven by the developmental genetic mechanisms invoked in limb bud development. Thus, we next investigated the expression pattern of some developmental genes that play roles in limb bud development by analyzing reporter transgenic fish lines.

**Fig. 5:**
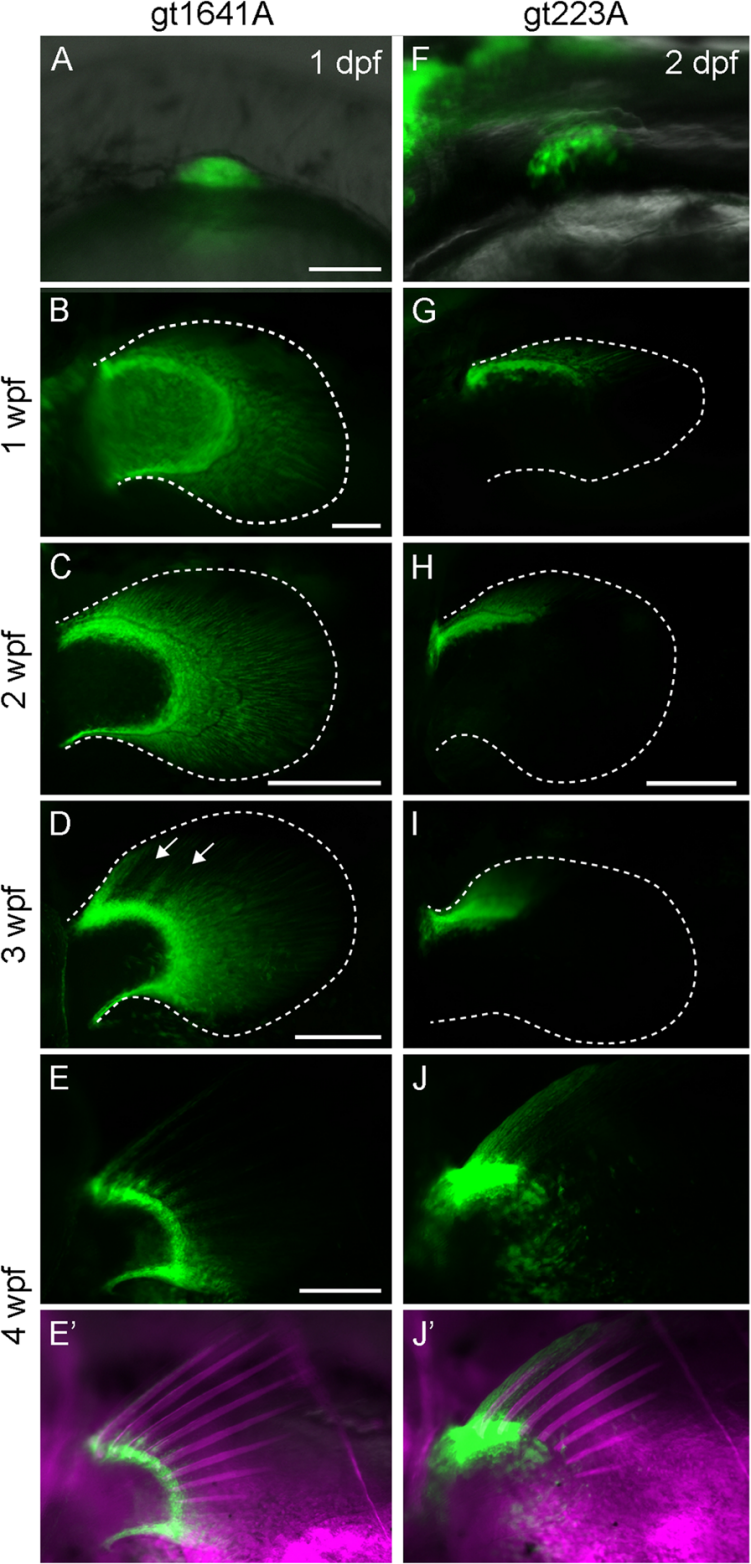
Expression pattern of EGFP in the pectoral fin of *gt1641A* and *gt223A* transgenic zebrafish. **a**-**e**’. Expression pattern of EGFP in the pectoral fin of a *gt1641A* transgenic fish observed at 1 dpf (**a**), 1 wpf (**b**), 2 wpf (**c**), 3 wpf (**d**), and 4 wpf (**e**, **e**’). **f**-**j**’. Expression pattern of EGFP in the pectoral fin of a *gt223A* transgenic fish observed at 2 dpf (**f**), 1 wpf (**g**), 2 wpf (**h**), 3 wpf (**i**), and 4 wpf (**j**, **j**’). White dashed lines indicate outline of the fin fold in the pectoral fin. Arrows in **d** indicate depletion of EGFP expression. Magenta in **e**’ and **j**’ indicate calcified bones stained by Alizarin Red. Scale bars in **a** and **b** and those in **c**, **d**, **e**, and **h** indicate 75 μm and 250 μm, respectively

To obtain transgenic fish lines with specific labeling of cell types in the fin, we performed a large-scale gene trap screen using *Tol2*-transposon-based gene trap constructs containing the engineered gal4 transcription factor *gal4ff* [23, 24, 33]. We generated two transgenic lines, *gt1641A* and *gt223A*, in which the reporter UAS:GFP is expressed in mesenchymal cells of the pectoral fin bud at 1 or 2 dpf (Fig. 5a, f). We analyzed the transposon integration sites in these transgenic lines by Southern blotting and inverse PCR. In *gt1641A*, the gene trap construct was integrated within the *lhx2b* gene (Additional file 3A), and the UAS:GFP reporter was found to be expressed in the entire fin bud mesenchymal cells (Fig. 5a). *lhx2b* was expressed in the entire fin bud mesenchymal cells at 1 and 2 dpf (Additional file 3C–F). Similarly, an ortholog of the *lhx2b* gene, *lhx2*, is expressed in the entire fin/limb bud mesenchyme in a basal actinopterygian, a polyodon, and tetrapods [19, 34–36]. In *gt223A,* the gene trap construct had integrated within the *lhx9* gene (Additional file 2B), and the UAS:GFP reporter was expressed in the anterior part of the fin bud at day 2 (Fig. 5f). *lhx9* was also expressed in the anterior fin bud mesenchyme at 2 dpf (Additional file 3G–J). *lhx9,* an ortholog of the *lhx9* gene, is expressed in the anterior fin/limb bud mesenchyme in a polyodon and tetrapods [19, 35, 37, 38]. These results suggest that the expression of GFP/Gal4 in these transgenic fish lines reproduces the expression pattern of these fin/limb developmental genes. *lhx2* and *lhx9* play a role in AP patterning in the limb bud under control of the Shh from the ZPA [19, 34–38]; thus, our reporter lines can be used to study whether the formation of fin rays and distal radials at a later larval stage is due to the AP patterning mechanism of limb/fin bud development.

We assessed the GFP expression pattern of these lines in the later development of fin rays and distal radials at 1, 2, 3, and 4 wpf. Continuous GFP expression was observed in fin bud cells in *gt1641A* larvae, with stronger GFP expression in the marginal mesenchymal cells and in mesenchymal cells inside the fin fold at 1 wpf (Fig. 5b). The inner mesenchymal cells with weaker GFP expression seemed to be in the endochondral disc [3, 4]. At 2 wpf, internal cells showed little GFP expression, but GFP expression was sustained in the marginal cells and mesenchymal cells of the fin fold (Fig. 5c). At later stages, 3 wpf and 4 wpf, GFP expression in the mesenchymal cells in the fin fold became gradually restricted posteriorly, except at the base of the fin ray (Fig. 5d–e’). The region where GFP expression had disappeared in the fin fold corresponded to the region where the fin ray formed. GFP expression in the mesenchymal cells in the marginal region was sustained until 4 wpf, with the most obvious expression in the posterior-most region (Fig. 5d–e’). Moreover, GFP expression in *gt223A* larvae was observed in the anterior marginal cells in the fin bud and contiguously anterior mesenchymal cells in the fin fold at 1 wpf (Fig. 5g). This expression pattern was sustained until 4 wpf (Fig. 5h–j’). GFP-expressing cells at 4 wpf covered the first fin ray and the region forming the first and second distal radials (Fig. 5JJ’). Anterior-restricted expression of the reporter GFP was also observed in other fins in *gt223A* larvae (data not shown). Since the first fin ray of fish fins generally showed specific morphology, such as non-bifurcated and thicker than other fin rays, *lhx9* may play a role in specific morphology in those fins, including pectoral fins.

The observations demonstrate that *lhx* genes exhibit region-specific expression in later larval stages when the fin rays and distal radials emerge, similar to that in the early developmental stages of fin buds. These results suggest that the formation of fin rays as well as distal radials is mediated by developmental mechanisms that employ developmental genes for limb/fin morphogenesis.

## Discussion

### Pattern of the pectoral fin ray and its variability along the anteroposterior axis based on the connection to distal radials

Our detailed morphological observations revealed that fin rays in the zebrafish pectoral fin can be divided into three parts based on the number of fin rays connected to the distal radials (Figs. 2, 3). The anterior part is in the domain that shows a one-to-one correspondence in fin ray-radial connection [3, 4], and the region contains five fin rays. Alternatively, the number varies in the middle and posterior domains. In the middle domain in which the sixth and seventh distal radials are located, one radial is connected to one or two fin rays, and there are totally three or four fin rays. The posterior domain has exhibits the greatest variation in the number of fin rays, two to five, which are connected to the eighth distal radial (though the eighth radial is occasionally missing). The fin rays in the zebrafish pectoral fin are not distributed randomly in the fin fold; they have a certain mode of connection to radials, suggesting that there is a skeletal pattern of fin rays along the AP axis. In this regard, zebrafish pectoral fin rays have a property similar to the endoskeletal domain of fins and limbs [1–3]. Interestingly, the variation in the number of fin rays does not randomly appear in a region, and the posterior domain is the main region where the number of fin rays varies.

Phenotypic variation is thought to be determined by both intrinsic genetic and non-genetic factors, such as developmental noise and environmental stimuli [39]. Our results reveal that the middle and posterior parts show a strain-specific difference in fin ray number variability (Fig. 3). Because laboratory strains of zebrafish have genetic variation that gives rise to phenotypic differences [31, 32], genetic differences among strains potentially affect the appearance of variability among fin ray numbers. Nonetheless, the IM-II strain, which is highly homogeneous compared to other strains [21], still shows variation in the number of fin rays, such as in the posterior part, as do other strains (Fig. 3). In addition, the numbers of rays were sometimes different on the left and right sides of the same individual (Fig. 2c). Thus, non-genetic factors also affect variation in the number of fin rays. In this regard, genetic and non-genetic factors play roles in different aspects of fin ray number variation. Genetic factors affect the tendencies in variability of fin ray number in the middle and posterior parts, while non-genetic factors affect the intra-strain variation, especially the variation in number of fin rays in the posterior part.

### Developmental process that mediates variability in pectoral fin ray number

Our observations of pectoral fin development showed that the difference between the regions showing a constant one-to-one aspect and variation in the number of fin rays may be due to alteration of the mode of distal radial localization (Fig. 4). The anterior several distal radials formed along the position of the fin rays, but posterior radials formed at the top of the proximal radials (Fig. 4). The alteration may result in a transition of the connection from one-to-one to one-to-many. Interestingly, some actinopterygian species have only one of these two localization modes. The pectoral fins in polyodonts and *Acipenser*, basal actinopterygians, show a morphological and developmental mode of one-to-many along the entire pectoral fin region [40–42]. In addition, the pectoral fin in *Polypterus*, another basal actinopterygian, has this morphology [43]. Alternatively, morphological and developmental features of the one-to-one mode have been reported in cichlid fishes [44, 45]. During median fin evolution in the actinopterygian lineage, the fin ray and distal radial connection reportedly shifted from a one-to-many to a one-to-one mode [46]. Thus, the one-to-many mode of the pectoral fin ray and distal radial connection is possibly an evolutionarily ancestral state in the actinopterygian lineage, and the features of fin ray and distal radial connection in zebrafish, with both modes observed along the AP axis, is an intermediate state.

As variation in the number of fin rays did not correlate with adult body size (Fig. 2), the number of fin rays was considered to be fixed at a certain stage of fin ontogeny. Indeed, the number of fin rays in our study was determined between 44 and 52 dpf (Fig. 4), indicating that the final number of fin rays depends on how many fin rays are added in the posterior region during this period, the latest stage of fin development. Interestingly, the last fin rays are added posteriorly, suggesting that one mechanism for posterior fin ray morphogenesis is sensitive to genetic/non-genetic factors that are responsible for the variability in the number of fin rays. Connections between fin rays and distal radials also varied. Because fin ray formation precedes distal radial formation (Fig. 4; see also [3]), variability in the fin ray-radial connection also appears to be a result of the process of fin ray number determination. Notably, the number of fin rays was mainly 12 or 13 in all of the strains we studied, and during the progression of fin ray formation, more time is required for formation of the 13th fin ray than for formation of the other fin rays (Fig. 4; Additional file 2). Overall, the fin ray number may be determined during formation of the 13th fin ray, and the mechanisms of the determination of fin ray number, including non-genetic factors, may affect fin ray development at the timing of formation of the 13th fin ray. In this regard, the determination of fin ray number may involve so-called self-organization mechanisms through the effect of non-genetic factors, such as alteration of the size of the fin ray formation field in the fin fold [47]. The posterior domain, which exhibits the greatest variability in the number of fin rays, may be a site of integration of self-organization and specific formation mechanisms.

### Developmental mechanism by which the pattern of the pectoral fin ray is formed along the AP axis

According to previous studies, some developmental genes, such as *hoxa13* and *hoxd13* genes, involved in patterning of the autopod and patterning along the AP axis in the limb bud are expressed in precursor cells of the fin ray and distal radials at an early larval stage of zebrafish pectoral fin development [15, 17]. These are also expressed in the paddlefish and catshark pectoral fin fold [18, 19], suggesting that these features of pattern formation are common and evolutionarily ancestral states in paired appendages of vertebrates. Considering these findings, we hypothesized that the patterned morphology of fin rays and their connection to radials in teleost fishes are mediated by fin/limb developmental genes during morphogenesis at a late larval stage. We thus investigated gene expression to test this hypothesis. Indeed, our observations of *lhx9* (*gt223A*) and *lhx2b* (*gt1641A*) reporter fish revealed that these genes are expressed in mesenchymal cells for both fin rays and distal radials until 4 wpf (Fig. 5), with certain patterns of expression. Taken together with the fact that *lhx9* and *lhx2* are also expressed in the paddlefish and catshark pectoral fin fold [18, 19], our results suggest that such fin rays and distal radial formation share some developmental mechanisms of patterning along the AP axis in fin development. Notably, reporter GFP expression of *gt1641A* was gradually restricted toward the posterior region as fin ray formation progresses (Fig. 5), indicating that the *lhx2b* gene is expressed in fin ray premature mesenchymal cells. The expression pattern of *lhx2*, the tetrapod ortholog of *lhx2b*, is regulated by Shh signaling from the ZPA, and *lhx2* reciprocally regulates *shh* expression for AP patterning and maintains the undifferentiated state of mesenchymal cells in the limb bud [35, 36]. In zebrafish and paddlefish, the *shh* gene is expressed in posterior mesenchymal cells of the developing fin fold region as well as in the early larval pectoral fin mesenchyme [19, 48]. Therefore, it is highly possible that the posterior signaling center with Shh signaling regulates the *lhx2b* expression pattern to retain the premature state of mesenchymal cells for fin ray and distal radial formation. In this sense, functional analysis of *lhx2b* would be interesting, and we are in fact carrying out such analysis. Additionally, considering that the variation in the number of fin rays is mediated by these posterior-organizing genes, it would be interesting to investigate whether these genes are sensitive to nongenetic factors.

## Conclusions

In this study, we found that zebrafish pectoral fin rays exhibit a pattern along the AP axis, which can be divided into three domains categorized by connection between fin rays and distal radials. This AP pattern in the fin ray-radial connection was confirmed by observation of their morphogenesis process. In addition, AP pattern formation in the fin ray-radial connection was found to be mediated by developmental genes for AP patterning in the limb/fin bud. Considering previous reports that fin ray precursor cells share some cellular and genetic properties with limb bud development, we suggest the possibility that the developmental mechanisms of fin rays and their connection are comparable to those of the distal element of the limb skeleton.

The posterior part of the three domains showed remarkable variability in the number of fin rays. Notably, non-genetic factors are thought to greatly affect intra-strain variation in the posterior domain. Our observation of the morphogenetic process of fin rays revealed that the number is at least partially determined by sensing of non-genetic factors during the process. In addition, the process of fin ray morphogenesis is mediated by developmental mechanisms, including *lhx* gene expression. Because *lhx* gene expression along the AP pattern in the limb bud is related to Shh signaling from the ZPA, it is possible that the posterior signaling center with Shh signaling regulates the *lhx2b* expression pattern for fin ray and distal radial formation and affects the variability of fin rays under the influence of nongenetic factors.

## Additional files

Additional file 1: Skeletal anatomy of the zebrafish pectoral fin at 21 dpf and 24 dpf. **A-A”.** Osteoblast cells (*sp7:mcherry*) and chondrogenic cells (*col2a:EGFP*) of the cranial region were observed at 21 dpf. **B-B″**. Magnified view of the pectoral fin in A-A”. **C-C″**. Osteoblast and chondrogenic cells of the pectoral fin at 24 dpf. Numbers in B and C indicate the fin rays and their order. Arrowheads with dr1–3 indicate the distal radials and their order. Scale bars in A, B, C indicate 500 μm, 200 μm and 200 μm, respectively. (TIF 4027 kb)

Additional file 2: Process of morphogenesis of the fin rays and radials from 24 dpf to 54 dpf. Calcified bones (Alizarin Red) and chondrogenic cells (*col2a:EGFP*) of the left pectoral fin in individual tracing specimens were observed at 24 dpf (**A**), 28 dpf (**B**), 32 dpf (**C**), 36 dpf (**D**), 40 dpf (**E**), 44 dpf (**F**), and 54 dpf (**G**). Orange arrowheads with numbers indicate the most posterior fin rays. White arrowheads with dr1–8 indicate newly appearing distal radials. The scale bar in A indicates 200 μm. CSZ, cartilage subdivision zone. sc, anlagen of the scapula. The asterisk in B indicates the reflected signal of the iridophore on the swim bladder. (TIF 4641 kb)

Additional file 3: Integration site of *gt1641A* and *gt223A* transgenic zebrafish. Structure of the insertion of the *Tol2*-transposon-based *gal4* gene trap cassette in the *lhx2b* locus in the *gt1641A* line (**A**) and *lhx9* locus in the *gt223A* line (**B**). Bending arrows indicate transcription start sites and orientations of transcription. White and black boxes indicate exons of untranslated and translated regions, respectively. Arrows on white boxes with black arrowheads in boxes at both ends indicate the *Tol2*-transposon-based *gal4* gene trap cassette, and the orientation of the arrows indicates the orientation of the gene trap and transcription of *gal4*. **C-E, G-I.** Expression pattern of *lhx2b* (C-E) and *lhx9* (G-H) observed in the pectoral fin bud (C, D, G, H) and the upper half of body (E, I) at 1 dpf (C, G) and 2 dpf (D, E, H, I). **F, J.** Expression pattern of EGFP in the upper half of body of *gt1641A* (F) and *gt223A* (J) transgenic fish observed at 2 dpf. Scale bases in C, E, and F indicate 100 μm, 200 μm, and 250 μm, respectively. (TIF 2045 kb)

## Data Availability

All data are available from the corresponding authors on request. Fish materials, including transgenic fish and specific wildtype strains, are available from KKa (for *gt223A*, *gt1641A*, *UAS:GFP*, AK, TAB), AK (for *sp7:mCherry*), MS (for IM-II), and GA (for *col2a1a:EGFP*).
