# Supplementary figures and images for: Pattern of fin rays along the antero-posterior axis based on their connection to distal radials
Source: Zoological Lett. 2019 Sep 18;5:30. doi: 10.1186/s40851-019-0145-z (PMC6751676; doi:10.1186/s40851-019-0145-z)

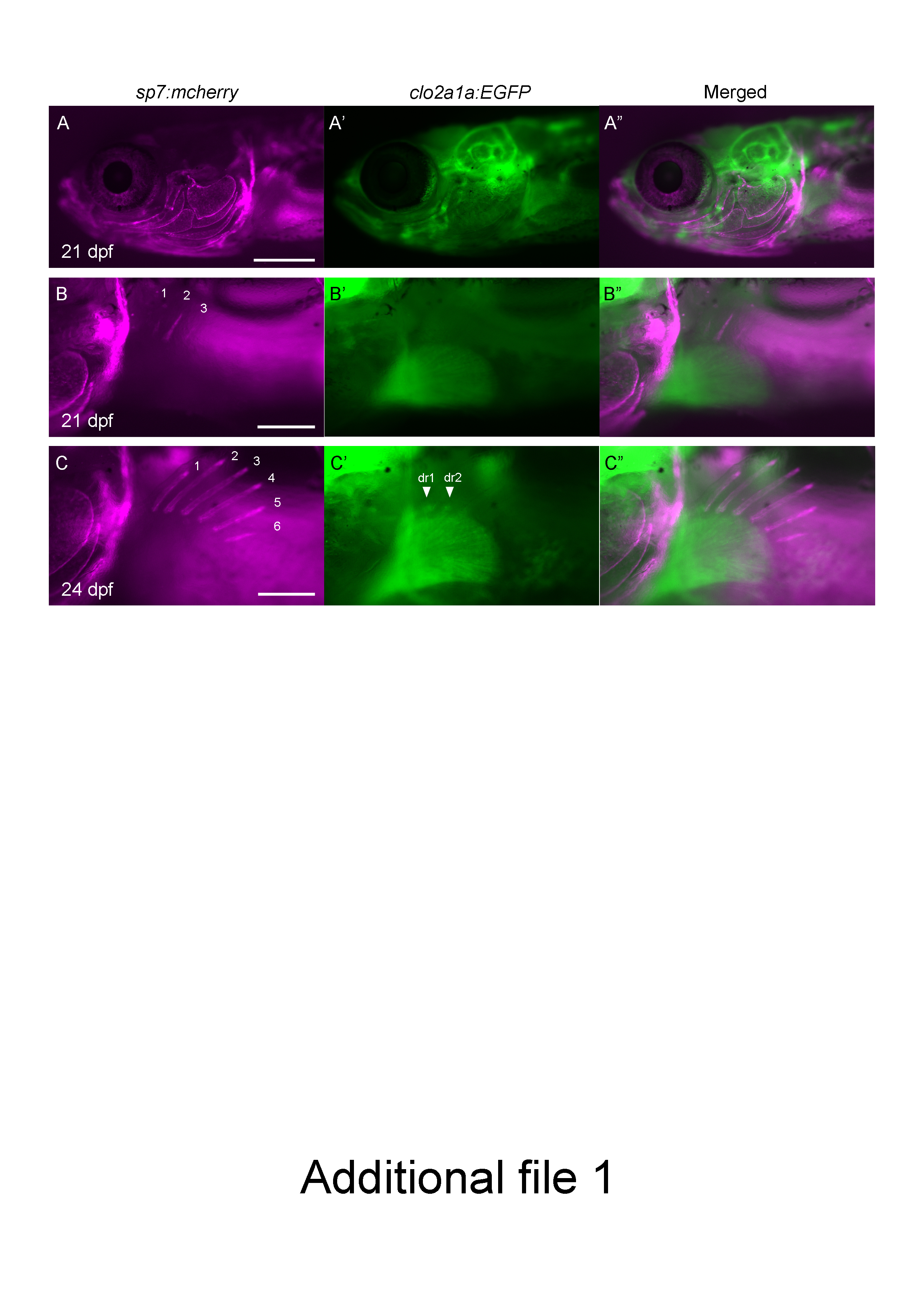

Supplement: Supplementary file 1 — Skeletal anatomy of the zebrafish pectoral fin at 21 dpf and 24 dpf. A-A”. Osteoblast cells (sp7:mcherry) and chondrogenic cells (col2a:EGFP) of the cranial region were observed at 21 dpf. B-B″. Magnified view of the pectoral fin in A-A”. C-C″. Osteoblast and chondrogenic cells of the pectoral fin at 24 dpf. Numbers in B and C indicate the fin rays and their order. Arrowheads with dr1–3 indicate the distal radials and their order. Scale bars in A, B, C indicate 500 μm, 200 μm and 200 μm, respectively. (TIF 4027 kb) [file 40851_2019_145_MOESM1_ESM.tif]

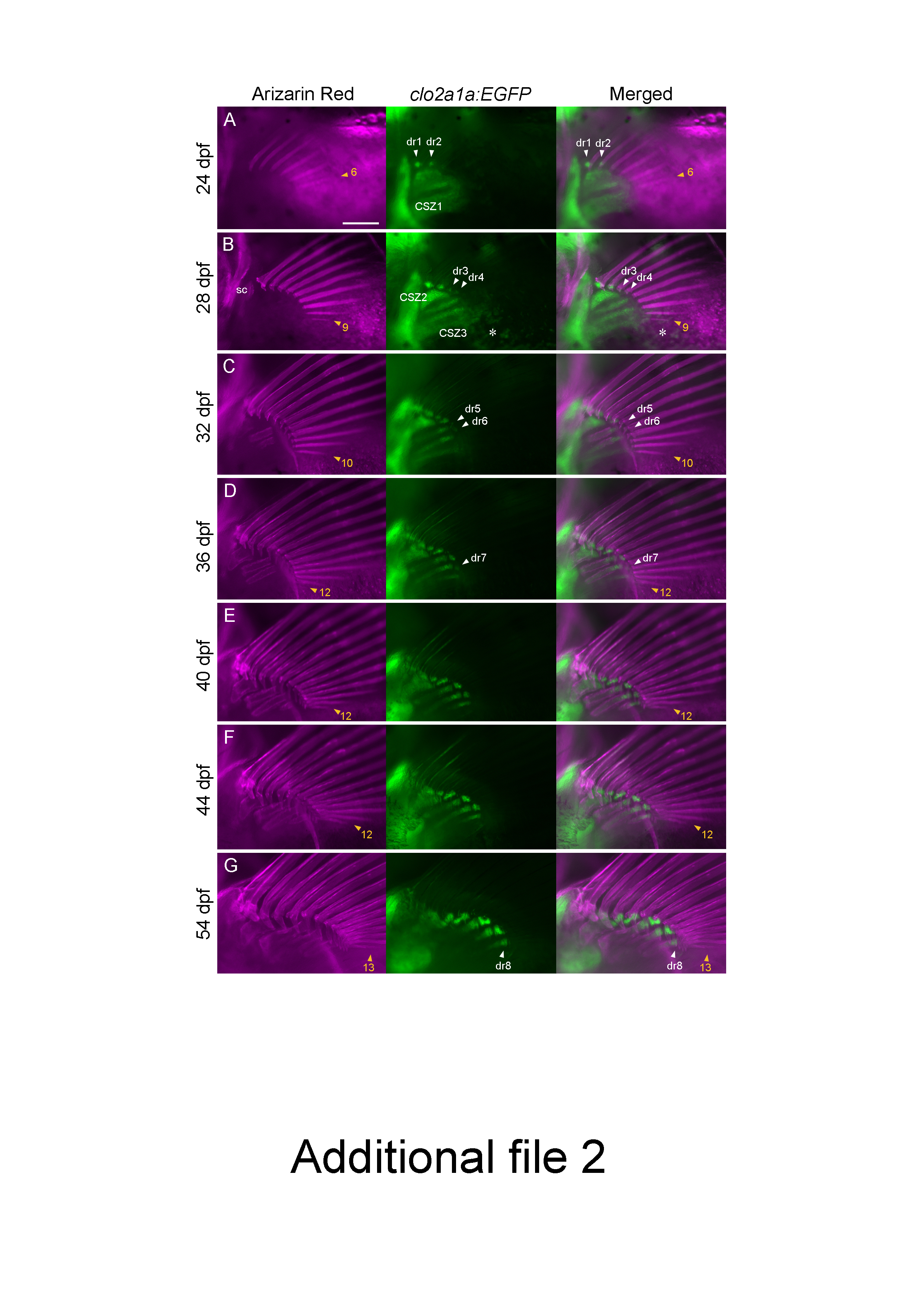

Supplement: Supplementary file 2 — Process of morphogenesis of the fin rays and radials from 24 dpf to 54 dpf. Calcified bones (Alizarin Red) and chondrogenic cells (col2a:EGFP) of the left pectoral fin in individual tracing specimens were observed at 24 dpf (A), 28 dpf (B), 32 dpf (C), 36 dpf (D), 40 dpf (E), 44 dpf (F), and 54 dpf (G). Orange arrowheads with numbers indicate the most posterior fin rays. White arrowheads with dr1–8 indicate newly appearing distal radials. The scale bar in A indicates 200 μm. CSZ, cartilage subdivision zone. sc, anlagen of the scapula. The asterisk in B indicates the reflected signal of the iridophore on the swim bladder. (TIF 4641 kb) [file 40851_2019_145_MOESM2_ESM.tif]

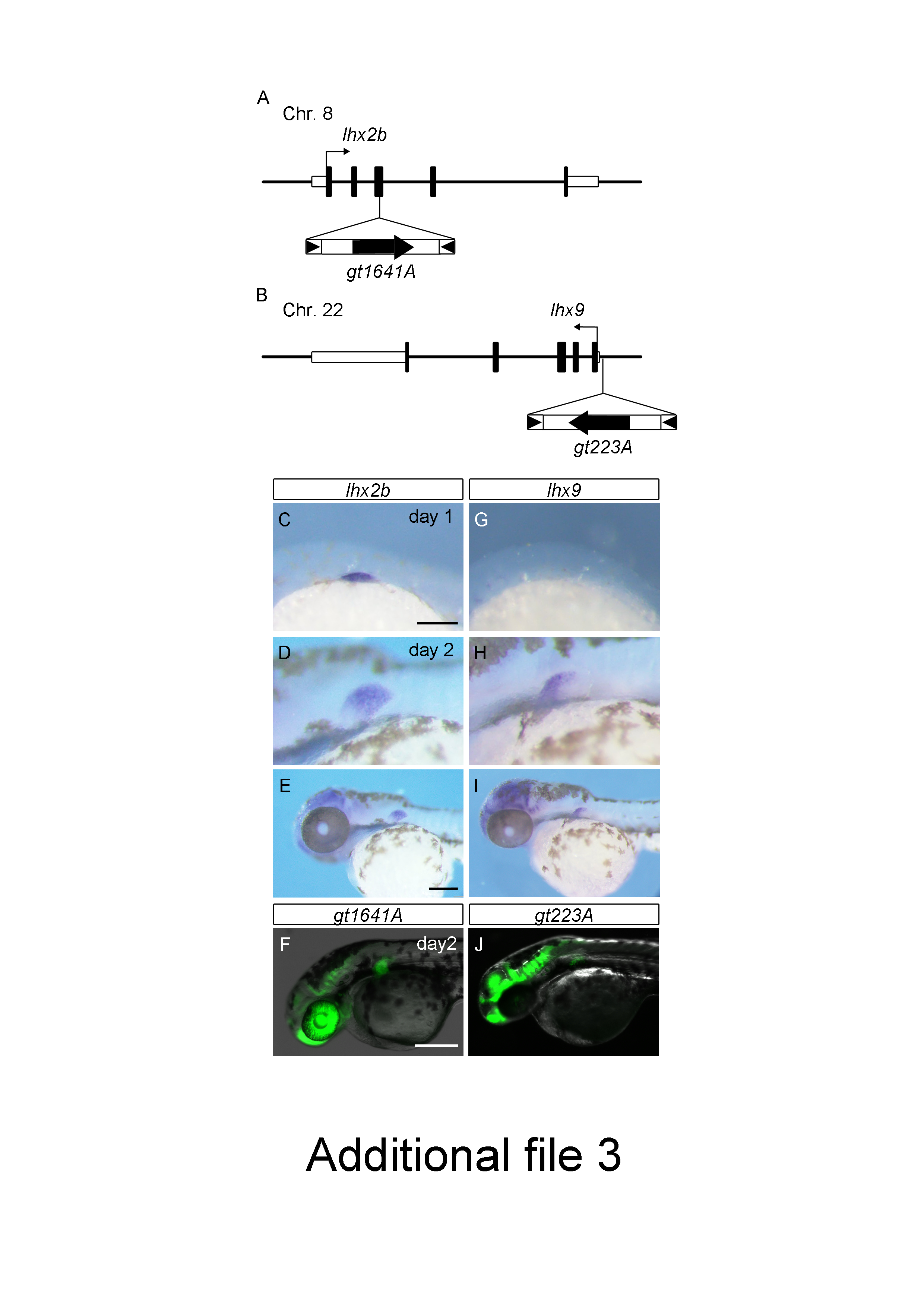

Supplement: Supplementary file 3 — Integration site of gt1641A and gt223A transgenic zebrafish. Structure of the insertion of the Tol2-transposon-based gal4 gene trap cassette in the lhx2b locus in the gt1641A line (A) and lhx9 locus in the gt223A line (B). Bending arrows indicate transcription start sites and orientations of transcription. White and black boxes indicate exons of untranslated and translated regions, respectively. Arrows on white boxes with black arrowheads in boxes at both ends indicate the Tol2-transposon-based gal4 gene trap cassette, and the orientation of the arrows indicates the orientation of the gene trap and transcription of gal4. C-E, G-I. Expression pattern of lhx2b (C-E) and lhx9 (G-H) observed in the pectoral fin bud (C, D, G, H) and the upper half of body (E, I) at 1 dpf (C, G) and 2 dpf (D, E, H, I). F, J. Expression pattern of EGFP in the upper half of body of gt1641A (F) and gt223A (J) transgenic fish observed at 2 dpf. Scale bases in C, E, and F indicate 100 μm, 200 μm, and 250 μm, respectively. (TIF 2045 kb) [file 40851_2019_145_MOESM3_ESM.tif]
